# Topical Immunotherapy in Alopecia Areata

**DOI:** 10.4103/0974-7753.66911

**Published:** 2010

**Authors:** Gurcharan Singh, MS Lavanya

**Affiliations:** Department of Dermatology and STD, Sri Devaraj Urs Medical College, Tamaka, Kolar – 563 101, India

**Keywords:** Alopecia Areata, dinitrochlorobenzene, squaric acid dibutylester

## Abstract

Alopecia Areata (AA) is a common non-scarring alopecia directed against the anagenic hair follicle. Various treatment modalities have been used for the treatment of severe AA. Topical immunotherapy is the best documented treatment so far for severe and refractory AA. Dinitrochlorobenzene (DNCB), squaric acid dibutylester (SADBE), and diphencyprone (DPCP) are the contact allergens used for this purpose. DNCB has been found to be mutagenic by the Ames test and is largely replaced by DPCP and SADBE. DPCP and SADBE are both known to be non-mutagenic compounds and have comparable efficacy results and relapse rates. SADBE requires special solvents and additives to maintain its potency and is more expensive than the rest. DPCP has a response rate varying from 60% in severe Alopecia Areata to 17% in patients with alopecia totalis or universalis, and shows about 88 to 100% high response rate in patients with patchy Alopecia Areata.

## INTRODUCTION

Alopecia areata (AA) is a common non-scarring alopecia directed against the anagenic hair follicle. It occurs in approximately 2% of the population. It is usually characterized by limited alopecic patches on the scalp. Various treatment modalities have been used for the treatment of AA. Topical therapies such as, corticosteroids, anthralin, minoxidil, contact sensitizers, and systemic therapies including steroids, cyclosporine, phototherapy, methotrexate, and dapsone are few of the options that have been tried. Topical immunotherapy is the best documented treatment so far for severe AA.

Topical immunotherapy is defined as the induction and periodic elicitation of allergic contact dermatitis (ACD) by applying a potent contact allergen.[1] In 1965, the alkylating agent triethyleneimino benzoquinone was the first topical sensitizer used to treat cutaneous disease, but it was abandoned on account of its mutagenicity. Later nitrogen mustard, poison ivy, nickel, formalin, and primin were tried, mainly as topical immunotherapy, for Alopecia Areata and warts. Contact immunotherapy was introduced in 1976, by Rosenberg and Drake. Later, potent contact allergerns namely dinitrochlorobenzene (DNCB), squaric acid dibutylester (SADBE), and diphencyprone (DPCP) superseded the earlier used allergens.[2]

There are several reports of the use of topical immunotherapy in the treatment of basal and squamous cell carcinoma, Bowen’s disease, actinic keratosis, cutaneous T-cell lymphoma, and primary and secondary malignant melanoma. Other conditions that have been treated succesfully include lichen nitidus, nodular prurigo, vulval intraepithelial neoplasms, vulval paget’s disease, condyloma accuminata, conjunctival squamous papillomata, atopic eczema, and systemic lupus erythematosus, where improvement of the immune parameter and its symptoms has been noted.[2] Their efficacy in chronic prurigo and atopic dermatitis is recognizable, where Th2 cells may play a pathogenic role.[3]

## DNCB

Dinitrochlorobenzene is a potent contact allergen that was found in 1912, and is used widely for induction of hair refractory in Alopecia Areata (AA) and inoculation of viral warts.[2] DNCB depletes the glutamine in rat skin and has strong mutagenic properties in the Salmonella enteritidis serotype, Salmonella typhimurium. It has been found to be mutagenic by the Ames test and genotoxic by the sister chromosomal exchange in human fibroblasts. Its fluoro derivatives promote tumor in mice.[2,4] It contains contaminants that are mutagenic and carcinogenic to animals.[4] It has a 65% absorption rate through the skin and 13% of the radiolabeled compound is recoverable in the urine. Excretion is primarly renal and serum half life in four hours.[2] It has been tried in human immunodefeciency virus (HIV) infection to raise the CD4 + T lymphocyte count and to decrease the number of opportunistic infections.[3] Although no evidence of carcinogenicity is reported in patients treated with DNCB, it has been largely replaced by DPCP and SADBE.[2]

## SADBE

Squaric acid dibutylester is a strong sensitizer, comparable to DNCB, and was found to be a potent contact allergen in 1979.[2,4] It is used in the industry as a stabilizer and anti-fog agent in photographic emulsion.[4]

It is considered as an ideal immunogen as it is found in the natural environment and is not known to cross react with chemicals.[5] It has been used for the treatment of AA and warts. It is unstable in acetone, unlike DPCP and requires special solvents and additives to maintain potency because of its tendency to undergo hydrolysis.[2] It is found to be non-mutagenic in the highly sensitive salmonella microsome test system.[4] SADBE is more expensive than other contact allergens.[2] No identifiable contaminants of its preparations have been discovered. Nevertheless, additional testing in animals is required to ascertain the mutagenicity and safety of SADBE, because of the occurrence of carcinogenesis reported in a few mice after its subcutaneous injection.[4] SADBE has been combined with topical dry ice, cryotherapy, carpronium chloride (a parasympathetic nerve stimulant) and / or oral cepharanthin (a biscoclaur alkaloid). Combination therapies are now considered to be a choice for refractory AA rather than topical SADBE alone.[6]

## DIPHENCYPRONE

Diphencyprone was first synthesized in 1959 and was prepared by bromination of dibenzylketone to form a precursor α- α’dibromodibenzyl ketone, which was cyclized with a base, to yeild DPCP. It is a potent contact allergen in humans and animals; 98 - 99% of the cases of Alopecia Areata can be sensitized on the scalp skin. Ultraviolet radiation and heat causes its degradration to diphenylacetylene and carbon monoxide. The standard solvent, acetone, is a strong UV light absorber and inhibits this process. DPCP is non-mutagenic in the Ames assay at concentrations of 50 and 100µg/ml^-1^. However α- α’dibromodibenzylketone is a potential contaminant and is mutagenic *in vitro*.[2] It was found that the proliferative responses of peripheral blood mononuclear cells (PBMC), obtained during and before DPCP application, showed a close association with hair re-growth responses to DPCP immunotherapy. In particular, poor clinical responses to topical immunotherapy can be expected in 70 ± 80% of AA patients who express lower PBMC responses than the control mean ± SD.[7]

Response rates range from 4 to 85% with DPCP and this wide range may be due to the differences in the number of patients, type, duration, and severity of AA, and the time of treatment and methods of evaluation undertaken in the various studies. A study showed that the response rate with DPCP varied from 60% in severe Alopecia Areata to 17% in patients with alopecia totalis or universalis, and showed about 88 to 100% high response rate in patients with patchy Alopecia Areata.[8] The immunohistochemical analysis of DPCP-treated mice models with hair loss, clinically and pathologically similar to AA, revealed reduction in the intrafollicular CD8 lymphocytes in the skin biopsy specimens.[9] In some patients, regrowth occured at sites distant from the site of application, or denser and faster growth was observed over the untreated site, termed as the ‘castling phenomenon’. A possible systemic effect of localized application was considered. It was observed after SADBE and DPCP treatment.[10]

## MECHANISM OF ACTION

Topical immunotherapy acts by varied mechanisms of action. Mainly a decrease in CD4 to CD8 lymphocyte count from 4:1 to 1:1 occurs.[2] A decrease in intrabulbar CD6 lymphocytes and langerhans cells is noted. Happle *et al*., proposed the concept of ‘Antigenic competition,’ where an allergic reaction generates suppressor T cells that non-specifically inhibits the autoimmune reaction against a hair follicle constituent. Expression of class I and II MHC molecules, which are normally decreased in areas affected by AA, disappear after topical immunotherapy treatment.[4] Significant nonspecific reduction in delayed type hypersensitivity reactions to unrelated antigens, occuring at remote sites, is reported. Contact allergens also tend to attract a new population of T cells in to the treated areas of the scalp, and thus enhancing a clearance of putative follicular antigen. A‘cytokine inhibitor’ theory has been planned, which proposes a possible interference of contact allergens with the pre-existing proinflammatory cytokines and also their continued production by follicular keratinocytes.[2] The other events noted are a decrease in the raised interferon γ levels, increase in mRNA expression of interleukins 2, 8, 10, and tumor necrosis factor α in the lesional skin.[1] It is also considered that interleukin 10 is responsible for the effectiveness of DPC, by inhibiting T lymphocytes. In succesfully treated patients, increased levels of the tumor growth factor β was detected.[2]

## METHOD OF SENSITIZATION

The scalp is the usual sensitization site. A cotton tipped applicator saturated with 2% DPCP in acetone is applied to an area of at least 10 cm^2^. Patients are advised to avoid washing the area and protect it from sunlight for 48 hours. Applications of the contact allergen are repeated weekly with increasing concentrations. The usual initial concentration of DPCP is 0.001 – 0.1% and this is repeated weekly, to induced a mild contact eczema, with concentration adjusted according to the response. An eczematous response (which may occur after five days or more) indicates that sensitization has taken place. A second application may be made if necessary. Only 1 - 2% of the patients fail to sensitize.[2]

## EFFICACY EVALUATION

The clinical response after six months of treatment was rated according to a grading system proposed by Mcdonald Hull and Norris:[11]

Grade 1 - Regrowth of vellus hair
Grade 2 - Regrowth of sparse pigmented terminal hair
Grade 3 - Regrowth of terminal hair with patches of alopecia
Grade 4 - Regrowth of terminal hair on scalp

### Non-responders

If no regrowth was observed within six months of treatment, the patient was considered to be a non- responder.[4] Evaluation of plucked hair was done using light microscopy, for evaluation of anagen/telogen ratio, and the presence of dystrophic hair on a scalp biopsy specimen demonstrated the pathological scarring pattern in a small fraction of populatio, predicting therapeutic failure.[11]

### Tolerance

It is defined as the absence of an immunological response to a foreign antigen.

Vander Steen *et al*., defined tolerance as the continous increase in the concentration of applied DPCP until a concentration of 2% was reached, with producing adequate dermatitis resulting in the loss of all regrown hair.[4]

## EFFICACY RESULTS

Total hair regrowth rates were 77% with DPCP, 64% with SADBE, 60% with minoxidil and 60% with placebo, with no statistically significant difference between them, in a study by Rokhsar *et al*.[4] Complete hair regrowth was 48% in a series of 52 patients studied.[11] Wiseman *et al*., reported 40% complete regrowth in 124 patients, and Van der steen *et al*., detected 50.4% regrowth in a study of 139 patients treated with DPCP.[12] In a study with a long follow-up period (mean 30.8 months) long-term maintenance of full regrowth, while on continuing treatment, was demonstrated by 38% of the responders and by 19% after goingoff all treatment.[13]

Wiseman *et al*.’s study with DPCP showed 100, 80, 60.3, and 17.4% for patients with 26 - 49%, 50 - 74%, 75 - 99% of AA and for patients of AA totalis/universalis, respectively.[11] The relapse rate was found to be about 62.6% with DPCP after 35 months of follow-up compared to 68.9% with SADBE.[14] Efficacy with DPCP was assessed in a study with four patients of ophiasis, who had been unresponsive to previous treatment. Three out of the four patients treated had grade 3 to grade 4 regrowth of hair after six months, with no significant side effects observed.[15] Tosti *et al*, reported complete hair regrowth to be 30.3% with SADBE.[16]

No statistically significant difference in regrowth of hair and relapse rate has been observed between DPCP and SADBE in the previous studies [Table 1].[4,14]

**Table 1:** Comparative efficacy of DPCP and SADBE therapy in Alopecia Areata

Contact sensitizer	Efficacy results[4] (%)	Relapse rate[14] (%)
Diphencyprone	77	62.6
Squaric acid dibutylester	64	68.9

Efficacy levels with DPCP therapy in various studies is shown below in Table 2

Efficacy levels with DPCP therapy in various studies is shown below in Table 2

**Table 2:** Efficacy levels with DPCP therapy in various studies

Study	DPCP - Complete hair regrowth (%)
Wiseman[12]	40
Van der steen[12]	50.4
Contellesa[11]	48
Sotiriadis[13]	38
Aghaei[14]	22.2

## PROGNOSTIC FACTORS

A less favorable response is seen with the type and extent of AA, long duration and disease before treatment, nail changes like piting, ridging, sandpaper nails, red lunula, and so on. Early development of contact dermatitis is a marker of a possible positive outcome. A more favorable clinical outcome may occur in patients in whom sensitization induces contact dermatitis within 10 weeks of application.[17]

In another study, a positive personal and family history of thyroid disease, atopy, diabetes mellitus, sex, age, type of AA, and duration of disease before treatment, had no significant correlation with the treatment response.[14] In a study done on 135 cases of AA treated with DPC, excellent response was seen in 100, 77, 54, 50, and 41% in Grade 1, Grade 2, Grade 3, AA totalis, and AA universalis patients, respectively.[18]

## SIDE EFFECTS

Topical immunotherapy can manifest few side effects such as persistent dermatitis, painful cervical lymphadenopathy, generalized eczema, blistering, contact leukoderma, and urticarial reaction. Systemic manifestations such as fever, arthralgia, and yellowish discoloration of gray hair are noted more often with DNCB.[4] Persistent contact dermatitis at the site of application and singular pigmentary metamorphosis observed in the newly grown scalp hair have been noted with the use of SADBE.[19] A case of discoid lupus erythematosus in which the condition was exacerbated by allergic contact dermatitis and induced by squaric acid dibutylester (SADBE) in Alopecia Areata, has been reported.[20] Dyschromia confetti is noted with the use of DPCP and suggests a possible absorption of it through the skin and distribution throughout the body, although it is reported not detecable in the serum or urine, after topical application.[2]
